# Paediatric Fever Management Practices and Antipyretic Use Among Doctors and Nurses in Australian Emergency Departments

**DOI:** 10.1111/1742-6723.70165

**Published:** 2025-11-03

**Authors:** Anastasia Mutic, Rui Zhi Tan, Eunicia Tan, Michael C. Fahey, Emily Callander, Libby Haskell, Shane George, Meredith L. Borland, Naomi Loftus, Jeremy Furyk, Natalie Phillips, Jane Bourke, Stuart R. Dalziel, Simon Craig, the PREDICT Network

**Affiliations:** ^1^ Department of Paediatrics Monash University Clayton Victoria Australia; ^2^ Department of Surgery, Faculty of Medical and Health Sciences The University of Auckland Auckland New Zealand; ^3^ Emergency Department Middlemore Hospital Auckland New Zealand; ^4^ Department of Paediatric Neurology Monash Health Clayton Victoria Australia; ^5^ School of Public Health University of Technology Sydney Sydney Australia; ^6^ Children's Emergency Department Starship Children's Hospital Auckland New Zealand; ^7^ Department of Paediatrics: Child and Youth Health, Faculty of Medical and Health Sciences The University of Auckland Auckland New Zealand; ^8^ Department of Emergency Medicine Gold Coast University Hospital Southport Queensland Australia; ^9^ School of Medicine and Dentistry Griffith University Southport Queensland Australia; ^10^ Child Health Research Centre The University of Queensland Brisbane Queensland Australia; ^11^ Emergency Department Perth Children's Hospital Nedlands Western Australia Australia; ^12^ Divisions of Emergency Medicine and Paediatrics, School of Medicine University of Western Australia Crawley Western Australia Australia; ^13^ Emergency Department Monash Medical Centre Clayton Victoria Australia; ^14^ University Hospital Geelong, Barwon Health Geelong Victoria Australia; ^15^ School of Medicine Deakin University Geelong Victoria Australia; ^16^ Emergency Department Queensland Children's Hospital Brisbane Queensland Australia; ^17^ Department of Pharmacology, Biomedicine Discovery Institute Monash University Clayton Victoria Australia; ^18^ Departments of Surgery and Paediatrics: Child and Youth Health, Faculty of Medical and Health Sciences The University of Auckland Auckland New Zealand

**Keywords:** antipyretics, child, emergency departments, febrile, fever

## Abstract

**Objectives:**

To examine variation in practice and adherence to international clinical guidelines for the management of fever among Australian Emergency Department (ED) clinicians.

**Methods:**

Cross‐sectional survey across 22 Australian EDs. Clinical vignettes were used to determine compliance with international best practice guidelines (use of antipyretic monotherapy to alleviate fever‐associated child distress) for paediatric fever treatment. Comparisons were made between specialist paediatric EDs and general (non‐specialist paediatric) EDs, and between medical and nursing staff.

**Results:**

Of 539 survey respondents (300 doctors, 239 nurses; overall response rate 65.9%), only 9.3% (50/539, 95% confidence interval [CI] 7.1%–12.0%) adhered to evidence‐based practice guidelines. Specialist paediatric ED clinicians demonstrated less than half the adherence of those from general EDs (5.4% [11/204] vs. 12.4% [38/307], difference −7.0%, 95% CI −11.7% to −1.9%). In a febrile settled child with normal hydration, the proportion of respondents who opted for antipyretics more than doubled in the context of elevated vital signs (40.4% [218/539] vs. 83.1% [44/539], difference −42.7%, 95% CI −46.8% to −38.2%). Nearly half of respondents (239/539, 46.8%, 95% CI 42.4%–51.2%) endorsed giving combined antipyretic therapy. In a febrile settled child, most participants would give antipyretics for temperature reduction (453/539, 84.0%, 95% CI 80.7%–86.9%) and for decreased fluid intake (468/539, 87.5%, 95% CI 84.4%–90.0%). Over one‐third (192/539, 36.0%, 95% CI 32.1%–40.2%) recommended using antipyretics for febrile convulsion prevention during the current illness.

**Conclusions:**

Fewer than 10% of Australian ED clinicians self‐report practice consistent with international consensus recommendations for paediatric fever management.

## 1 Introduction

Fever is a common reason for families to seek medical care, and is present in approximately 15%–25% of paediatric consultations in primary care and emergency departments (EDs) [1, 2].

A normal response to infection, fever enhances the immune system's defences against pathogens [3]. Despite its protective purposes, a febrile episode in a child remains a major source of anxiety among caregivers and healthcare professionals [4]. This ‘fever phobia’ stems from false beliefs predominantly surrounding the neurological dangers of untreated fever, which include blindness, brain damage and even death [5]. This can lead to unnecessary medical visits and aggressive, unwarranted antipyretic practices that can result in medication‐induced toxicity due to overzealous administration or dosing errors [6, 7].

Current international guidelines from the UK's National Institute for Health and Care Excellence (NICE) [8], the American Academy of Pediatrics (AAP) [3] and the Italian Pediatric Society (IPS) [9] collectively recommend the use of a single agent (paracetamol or ibuprofen), based on their well‐documented safety and efficacy profiles [10], to alleviate a child's distress rather than for the singular objective of reducing body temperature. These guidelines also uniformly discourage the use of combined antipyretics, citing concerns over potential adverse effects [3, 8, 9]. Despite this, overuse of antipyretic administration remains a widespread practice among ED clinicians [11, 12], which may reinforce parental misconceptions and contribute to excessive, hazardous antipyretic use [3, 13].

Inconsistencies in paediatric fever management practices in the ED setting have recently been assessed in New Zealand, with a focus on children aged < 2 years [12, 14]. However, the impact of varying levels of paediatric specialisation on ED clinical practice is underexplored [15]. This paper aims to evaluate ED clinicians' self‐reported management of febrile children < 2 years of age across specialist paediatric and general EDs in Australia.

## 2 Methods

### 2.1 Design, Setting and Participants

We conducted a cross‐sectional survey, previously utilised in New Zealand [12], of ED doctors and nurses across 22 Australian EDs within the Paediatric Research in Emergency Departments International Collaborative (PREDICT) Network [16]. Eligible participants were full‐ or part‐time ED doctors and nurses (at all levels of training) who worked a minimum of one shift per week in an ED managing children < 2 years of age. We excluded those not currently active in clinical practice, junior medical staff rotating through ED placements, medical locum tenens, temporary nursing agency personnel and medical or nursing students.

An expression of interest, followed by an invitation to participate was emailed to each nominated PREDICT site representative.

### 2.2 Survey Instrument and Distribution

The survey was structured into four sections: participant information and consent, demographics, fever management and antipyretic use and factors impacting these clinical practices [12] (Appendix S1). Fever management practices and adherence to clinical practice guidelines were assessed using eight clinical vignettes and two questionnaire items [12].

Participants accessed and completed the survey anonymously via an online link disseminated by local site representatives. The survey, requiring 10–15 min for completion, was accessible for 8 weeks at each site. Responses were automatically and anonymously captured using the Research Electronic Data Capture (REDCap) electronic database system [17] hosted on Monash University servers, with access restricted to authorised research personnel. The study was deemed negligible risk and exempt from full ethics review by Monash Health Research Support Services (RES‐23‐0000‐780Q).

### 2.3 Outcomes

The primary outcome was adherence to international guideline recommendations concerning antipyretic administration in febrile children. Adherence was defined as *single* antipyretic therapy for relief of *discomfort*, rather than for the sole purpose of temperature reduction. Secondary outcomes included the use of alternating or combined antipyretic regimens; antipyretic use for the purpose of temperature reduction, reduced fluid intake or febrile convulsion prevention; utilisation of specific clinical practice guidelines and patient information sheets; and the impact of abnormal vital signs on antipyretic use.

### 2.4 Data Analysis

Survey data was analysed using IBM SPSS Statistics 29. ED types were grouped according to the Australasian College for Emergency Medicine (ACEM) Site Classification framework [18]. Categorical data were summarised using descriptive statistics and reported as count, percentages and associated 95% confidence intervals (CIs). Comparison of categorical variables was conducted via Pearson Chi‐squared tests. Absolute difference between doctors and nurses, and specialist paediatric and non‐specialist paediatric settings, was assessed using a two‐sample *z*‐test of proportions.

## 3 Results

Between March and May 2024, the survey was distributed to 818 ED clinicians (344 doctors, 305 nurses, 169 clinician types not recorded at the site level), of whom 551 completed the survey. Of these, 12 were excluded; 10 did not provide data for the primary outcome, and 2 did not report their profession. A total of 539 participants (300 doctors, 239 nurses) were included in the analysis, yielding an overall response rate of 65.9% (Table 1). The survey included representation from 22 Australian EDs, comprising 7 specialist paediatric and 15 general ED sites. Clinicians from Victoria, Western Australia and Queensland made up nearly three quarters of all responses.

**TABLE 1:** Participant characteristics.

	All participants		Doctors		Nurses	
	*N*	*n* (%)	*N*	*n* (%)	*N*	*n* (%)
State/territory	511		289		222	
Victoria		140 (27.4)		68 (23.5)		72 (32.4)
Queensland		124 (24.3)		87 (30.1)		37 (16.7)
Western Australia		117 (22.9)		54 (18.7)		63 (28.4)
New South Wales		55 (10.8)		28 (9.7)		27 (12.2)
Northern Territory		34 (6.7)		14 (4.8)		20 (9.0)
Tasmania		24 (4.7)		21 (7.3)		3 (1.4)
Not specified		9 (1.8)		9 (3.1)		0 (0)
South Australia		8 (1.6)		8 (2.8)		0 (0)
ED type (ACEM designation)^a^	511		289		222	
Major referral		255 (49.9)		154 (53.3)		101 (45.5)
Urban district		104 (20.4)		49 (17.0)		55 (24.8)
Regional referral/other		152 (29.7)		86 (29.8)		66 (29.7)
ED specialisation^a^	511		289		222	
Paediatric ED^b^		204 (39.9)		109 (37.7)		95 (42.8)
General ED		307 (60.1)		180 (62.3)		127 (57.2)
Years of professional experience^a^	538		300		238	
0–4		65 (12.1)		19 (6.3)		46 (19.3)
5–9		134 (24.9)		73 (24.3)		61 (25.6)
10–14		153 (28.4)		88 (29.3)		65 (27.3)
≥ 15		186 (34.6)		120 (40.0)		66 (27.7)
Clinical role—senior^c^	539	291 (54.0)	300	185 (61.7)	239	106 (44.4)
Paediatric‐specific qualifications^a^, ^d^	538	159 (29.6)	299	96 (32.1)	239	63 (26.4)

Abbreviations: ACEM, Australasian College for Emergency Medicine; ED, emergency department; *n*, number of respondents who selected a specific option; *N*, total number of respondents to the question.

^a^ Data were missing for the following questions: ED type (*n* = 28), ED specialisation (*n* = 28), years of experience in profession (*n* = 1) and paediatric‐specific qualifications (*n* = 1).

^b^ ED type and designation based on ACEM classification. Paediatric ED includes specialist and non‐specialist (co‐located but separately accredited paediatric ED). General ED refers to an ED not designated a paediatric ED.

^c^ Senior doctor role includes consultant, fellow; senior nurse role includes advanced practice nurse (nurse practitioner and nurse specialist), nurse educator, charge nurse.

^d^ Includes Fellowship in Paediatrics, subspecialty ACEM training in paediatric emergency medicine, Nursing Masters.

Among participants who indicated their primary hospital, half worked at a major referral centre (255/511, 49.9%) and over half were employed in general ED settings (307/511, 60.1%). Compared to nurses, a smaller proportion of doctors reported < 5 years of professional experience (19/300, 6.3% vs. 46/238, 19.3%) and a greater percentage of doctors held a senior clinical position (185/300, 61.7% vs. 106/239, 44.4%). More than one quarter of participants (159/538, 29.6%) held paediatric‐specific qualifications.

### 3.1 Primary Outcome

Overall, only 9.3% of respondents (50/539, 95% CI 7.1%–12.0%) reported practice consistent with guidelines advising antipyretic monotherapy in the presence of patient distress (Table 2). Notably, clinicians practising in specialist paediatric EDs demonstrated less than half the adherence rate of their counterparts in general EDs (5.4% vs. 12.4%, difference −7.0%, 95% CI −11.7% to −1.9%) (Table 3). However, they were more likely to report using clinical practice guidelines, providing patient information handouts and less likely to recommend antipyretics to prevent a febrile convulsion.

**TABLE 2:** Fever management practices by emergency department doctors and nurses.

	All participants		Doctors		Nurses		Percentage difference between doctors and nurses	*p*
	*N*	*n* (%, 95% CI)	*N*	*n* (%)	*N*	*n* (%)	% difference (95% CI)	
Clinical vignettes								
Adherence to best practice guidelines	539	50 (9.3, 7.1–12.0)	300	29 (9.7)	239	21 (8.8)	0.9 (−4.2 to 5.8)	0.73
Antipyretic use for temperature reduction	539	453 (84.0, 80.7–86.9)	300	270 (90.0)	239	183 (76.6)	13.4 (7.0–19.8)	< 0.01
Antipyretic use for reduced fluid intake^a^	535	468 (87.5, 84.4–90.0)	299	281 (94.0)	236	187 (79.2)	14.7 (8.8–20.6)	< 0.01
Combined antipyretic use^a^	511	239 (46.8, 42.4–51.2)	289	138 (47.8)	222	101 (45.5)	2.3 (−6.5 to 10.9)	0.61
Uses clinical practice guidelines^a^	531	304 (57.3, 53.0–61.4)	295	127 (43.1)	236	177 (75.0)	−31.9 (−39.6 to −23.8)	< 0.01
Type of clinical practice guidelines used^b^								
No guideline selected		235 (43.6, 39.4–47.9)		173 (57.8)		62 (25.9)	31.9 (23.8–39.6)	< 0.01
Local hospital		155 (28.8, 25–32.8)		36 (12)		119 (49.8)	−37.8 (−45.1 to −30.5)	< 0.01
State		49 (9.1, 6.8–11.8)		32 (10.7)		17 (7.1)	3.6 (−1.2 to 4.8)	< 0.01
Royal Children's Hospital, Melbourne		195 (36.2, 32.1–40.4)		88 (29.3)		107 (44.8)	−15.5 (−23.6 to −7.3)	0.11
National Institute for Health and Care Excellence		8 (1.5, 0.6–2.9)		4 (1.3)		4 (1.7)	−0.3 (−2.4 to 1.7)	0.63
Other^c^		12 (2.2, 1.2–3.9)		3 (1)		9 (3.7)	−2.7 (−5.4 to 0.1)	0.23
Gives patient information sheet at discharge^a^	534	385 (72.1, 68.1–75.7)	296	222 (75.0)	238	163 (68.5)	6.5 (−1.2 to 14.2)	0.10
Type of patient information sheet given^b^								
No information sheet selected		154 (28.5, 24.8–32.6)		78 (26)		76 (31.8)	−5.8 (−13.5 to 1.9)	0.16
Local hospital		146 (27.1, 23.4–31.1)		75 (25)		71 (29.7)	−4.7 (−12.3 to 2.9)	0.24
State		69 (12.8, 10.1–15.9)		54 (18)		15 (6.3)	11.7 (6.4–17)	< 0.01
Paediatric Improvement Collaborative, hosted by the Royal Children's Hospital, Melbourne website		250 (46.4, 42.1–50.7)		142 (47.3)		108 (45.2)	2.1 (−6.3 to 10.6)	0.64
Better Health Channel		12 (2.2, 1.2–3.9)		5 (1.7)		7 (2.9)	−1.2 (−3.8 to 1.3)	0.26
Other^c^		22 (4.1, 2.6–6.1)		12 (4)		10 (4.2)	−0.2 (−3.5 to 3.2)	0.76

Abbreviations: CI, confidence interval; *n*, number of responses that selected the specific outcome; *N*, total number of responses to the question; *p*, two‐tailed *p* value calculated with two proportion *z*‐test.

^a^ Data were missing for the following questions: antipyretic use for fluid reduction (*n* = 4), combined antipyretic used (*n* = 28), use of clinical practice guidelines (*n* = 8), gives patient information sheet (*n* = 5).

^b^ Participants can choose more than one option.

^c^ Other clinical practice guidelines include American Academy of Paediatrics, Perth Children's Hospital, Queensland Children's Hospital; other patient information sheets include Patient.info, UpToDate.

**TABLE 3:** Comparing fever management practices by paediatric ED and general ED clinicians.

	Specialist paediatric ED		General (not exclusively paediatric) ED		Percentage difference between Specialist paediatric and general ED	*p*
	*N*	*n* (%)	*N*	*n* (%)	% difference (95% CI)	
Adherence to best practice guidelines	204	11 (5.4)	307	38 (12.4)	−7.0 (−11.7 to −1.9)	0.01
Antipyretic use for temperature reduction	204	170 (83.3)	307	260 (84.7)	−1.4 (−8.0 to 5.1)	0.68
Antipyretic use for reduced fluid intake^a^	202	175 (86.6)	305	269 (88.2)	−1.6 (−7.6 to 4.3)	0.60
Antipyretic use for febrile convulsions^a^	201	58 (28.9)	304	121 (39.8)	−10.9 (−19.1 to −2.5)	0.01
Combined antipyretics	204	89 (43.6)	307	150 (48.9)	−5.2 (−13.9 to 3.6)	0.25
Alternating antipyretics^a^	199	144 (72.4)	303	212 (70.0)	−2.4 (−10.4 to 5.8)	0.56
Uses clinical practice guidelines^a^	201	125 (62.2)	303	161 (53.1)	9.1 (0.2–17.7)	0.05
Gives patient information sheets^a^	202	162 (80.2)	304	201 (66.1)	14.1 (6.2–21.5)	< 0.01

*Note:* Antipyretic use includes single, alternating and combination treatment.

Abbreviations: CI, confidence interval; *n*, number of responses that selected the specific outcome; *N*, total number of responses from each emergency department type; *p*, two‐tailed *p* value calculated with two proportion *z*‐test.

^a^ Data missing for the following questions: reduced fluid intake (*n* = 4), febrile convulsions (*n* = 6), alternating antipyretics (*n* = 9), uses clinical practice guidelines (*n* = 7), gives patient information sheets (*n* = 5).

Table 4 presents participants' responses to clinical vignettes with varying levels of patient distress, fluid intake and vital signs.

**TABLE 4:** Antipyretic use based on 6‐month‐old febrile clinical vignette patient with variable levels of discomfort, fluid intake and vital signs.

Clinical vignette	Vital signs				Percentage difference between normal and elevated vital signs	*p*
	Normal HR and RR		Elevated HR and RR			
	*N*	*n* (%, 95% CI)	*N*	*n* (%, 95% CI)	% difference (95% CI)	
Settled, drinking usual amount	539		539			
Give antipyretics		218 (40.4, 36.4–44.6)		448 (83.1, 79.7–86.1)	−42.7 (−46.8 to −38.2)	< 0.01
Give a *single* antipyretic (paracetamol or ibuprofen)		163 (30.2, 26.5–34.2)		277 (51.4, 47.2–55.6)	−21.2 (−26.3 to −15.9)	< 0.01
Give *both* paracetamol and ibuprofen at the same time		55 (10.2, 7.9–13.1)		171 (31.7, 27.9–35.8)	−21.5 (−25.1 to −17.8)	< 0.01
Paracetamol and/or ibuprofen not needed at this time		321 (59.6, 55.4–63.6)		91 (16.9, 13.9–20.3)	42.7 (38.3–47.0)	< 0.01
Settled, drinking 2/3 usual amount^a^	538		535			
Give antipyretics		388 (72.1, 68.2–75.7)		454 (84.9, 81.6–87.7)	−12.5 (−15.8 to −9.2)	< 0.01
Give a *single* antipyretic (paracetamol or ibuprofen)		287 (53.3, 49.1–57.5)		290 (54.2, 50.0–58.4)	−0.7 (−5.4–3.9)	0.38
Give *both* paracetamol and ibuprofen at the same time		101 (18.8, 15.7–22.3)		164 (30.7, 26.9–34.7)	−11.8 (−15.3 to −8.2)	< 0.01
Paracetamol and/or ibuprofen not needed at this time		150 (27.9, 24.3–31.8)		81 (15.1, 12.3–18.4)	12.5 (9.1–15.9)	< 0.01
Crying intermittently, drinking usual amount	539		539			
Give antipyretics		441 (81.8, 78.3–84.9)		482 (89.4, 86.5–91.8)	−7.6 (−10.4 to −4.8)	< 0.01
Give a *single* antipyretic (paracetamol or ibuprofen)		339 (62.9, 58.7–66.9)		295 (54.7, 50.5–58.9)	8.2 (3.9–12.4)	< 0.01
Give *both* paracetamol and ibuprofen at the same time		102 (18.9, 15.8–22.5)		187 (34.7, 30.8–38.8)	−12.8 (−16.4 to −9.1)	< 0.01
Paracetamol and/or ibuprofen not needed at this time		98 (18.2, 15.1–21.7)		57 (10.6, 8.2–13.5)	7.6 (4.8–10.4)	< 0.01

Abbreviations: CI, confidence interval; HR, heart rate; *n*, number of responses that selected the specific outcome; *N*, total number of responses to the question; *p*, two‐tailed *p* value calculated with paired sample two proportion *z*‐test; RR, respiratory rate.

^a^ Data missing for the following questions: normal vital signs: settled, drinking 2/3 usual amount (*n* = 1), elevated vital signs: settled, drinking 2/3 usual amount (*n* = 4).

In the vignette depicting a febrile 6‐month‐old child without discomfort and normal hydration, the percentage of respondents opting to administer antipyretics more than doubled when elevated vital signs were introduced (40.4% vs. 83.1%, difference −42.7%, 95% CI −46.8% to −38.2%).

In the vignette where the child exhibited intermittent crying, single antipyretic use remained the most frequent response across both normal and abnormal vital signs. However, when vital signs were elevated, a smaller proportion of participants were inclined to give antipyretic monotherapy (62.9% vs. 54.7%, difference 8.2%, 95% CI 3.9%–12.4%).

Overall, practice consistent with clinical recommendations declined in the context of elevated vital signs, despite the same levels of patient discomfort and fluid intake. Additionally, more doctors than nurses were inclined to give antipyretics across all clinical vignettes, regardless of the child's distress level (Tables S1 and S2).

### 3.2 Secondary Outcomes

#### 3.2.1 Combined and Alternating Antipyretic Use

Approximately half of the respondents endorsed the use of combined antipyretic therapy in response to the vignettes (Table 2). Preferences for the combined regimen approach significantly increased in the scenario of a febrile but settled infant with elevated vital signs (10.2% [55/539] vs. 31.7% [171/539], % difference = −21.5%, 95% CI −25.1% to −17.8%) (Table 4). A similar inclination was noted in the case of a febrile child who was intermittently distressed. Across all clinical scenarios, a greater proportion of doctors compared to nurses advocated combined antipyretics, irrespective of the child's observed discomfort level (Tables S1 and S2).

Nearly three‐quarters of participants favoured alternating antipyretic use when managing a febrile but settled 6‐month‐old infant with a viral illness, with equal proportions of doctors and nurses supporting this treatment strategy (Table 5b).

**TABLE 5:** Discharge advice regarding fever management by emergency department doctors and nurses.

	All participants		Doctors		Nurses		Percentage difference between doctors and nurses	*p*
	*N*	*n* (%, 95% CI)	*N*	*n* (%, 95% CI)	*N*	*n* (%, 95% CI)	% difference (95% CI)	
(a) Proportion of participants who would give antipyretics for febrile convulsion								
Prevention of febrile convulsions during current illness^a^	533		296		237			
Give medications to prevent febrile convulsions		192 (36.0, 32.1–40.2)		66 (22.3, 17.9–27.4)		126 (53.2, 46.8–59.4)	−30.9 (−38.6 to −22.7)	< 0.01
Regularly		41 (7.7, 5.7–10.3)		19 (6.4, 4.1–9.9)		22 (9.3, 6.2–13.7)	−2.9 (−7.6 to 1.8)	0.22
As needed if there is a fever		151 (28.3, 24.7–32.2)		47 (15.9, 12.1–20.5)		104 (43.9, 37.7–50.2)	−28.0 (−35.4 to −20.3)	< 0.01
Paracetamol and/or ibuprofen not needed to prevent febrile convulsions		341 (64.0, 59.8–67.9)		230 (77.7, 72.6–82.1)		111 (46.8, 40.6–53.2)	30.9 (22.7–38.6)	< 0.01
Management of fever during current illness^a^	530		294		236			
Give medications regularly to prevent fever		25 (4.7, 3.2–6.9)		13 (4.4, 2.5–7.5)		12 (5.1, 2.8–8.8)	−0.7 (−4.5 to 3.0)	0.72
Give medications as needed if there is a fever		67 (12.6, 10.1–15.8)		29 (9.9, 6.9–13.8)		38 (16.1, 11.9–21.4)	−6.2 (−12.0 to −0.4)	0.03
Give medications as needed if there is a fever and the infant is distressed		228 (43.0, 38.9–47.3)		131 (44.6, 39.0–50.3)		97 (41.1, 35.0–47.5)	3.5 (−5.0 to 11.9)	0.42
Give medications as needed if the infant is distressed, regardless of fever		207 (39.1, 35.0–43.3)		118 (40.1, 34.7–45.8)		89 (37.7, 31.8–44.0)	2.4 (−5.9 to 10.7)	0.57
Paracetamol and/or ibuprofen not needed at this time		3 (0.6, 0.1–1.7)		3 (1.0, 0.2–3.1)		0 (98.1–100)	1.0 (−0.6 to 2.5)	0.12
(b) Proportion of participants who would give antipyretics for fever secondary to viral illness								
Management of fever during current illness^a^	528		295		233			
Give paracetamol or ibuprofen, repeating doses of the same medication		87 (16.5, 13.5–19.9)		54 (18.3, 14.3–23.1)		33 (14.2, 10.2–19.3)	4.1 (−2.3 to 10.4)	0.20
Give alternating doses of paracetamol and ibuprofen		376 (71.2, 67.2–74.9)		210 (71.2, 65.8–76.1)		166 (71.2, 65.1–76.7)	−0.1 (−7.8 to 7.7)	0.99
Give both paracetamol and ibuprofen at the same time		43 (8.1, 6.1–10.8)		25 (8.5, 5.8–12.3)		18 (7.7, 4.9–11.9)	0.7 (−4.1 to 5.4)	0.76
Paracetamol and/or ibuprofen not needed at this time		22 (4.2, 2.7–6.3)		6 (2.0, 0.8–4.5)		16 (6.9, 4.2–10.9)	−4.8 (−8.6 to −1.1)	0.01

Abbreviations: CI, confidence interval; *n*, number of responses that selected the specific outcome; *N*, total number of responses to the question; *p*, two‐tailed *p* value calculated with two proportion *z*‐test.

^a^ Data missing for the following questions: Prevention of febrile convulsions during current illness (*n* = 6), management of fever during current illness (*n* = 9), management of fever secondary to viral illness (*n* = 11).

#### 3.2.2 Antipyretic Use for Temperature Reduction and Reduced Fluid Intake

Most participants reported administering antipyretics for temperature reduction and for decreased fluid intake in the context of normal vital signs (Table 2). In a febrile but settled infant (Table 4), over one‐third of respondents indicated antipyretic use for temperature reduction while the majority would use antipyretics to address reduced fluid intake.

Compared to nursing staff, a greater percentage of doctors reported antipyretic use for temperature reduction (270/300, 90.0% vs. 183/239, 76.6%; % difference 13.4%, 95% CI 7.0%–19.8%) or reduced fluid intake (281/299, 94.0% vs. 187/236, 79.2%; % difference 14.7%, 95% CI 8.8%–20.6%) (Table 2).

#### 3.2.3 Use of Clinical Practice Guidelines and Patient Information Sheets

More than half of participants reported the use of clinical practice guidelines (CPGs) and nearly three‐quarters reported routine provision of patient information sheets to caregivers upon discharge (Table 2). The most common source of guidelines and information sheets was the Paediatric Improvement Collaborative hosted by Royal Children's Hospital (Melbourne).

Compared to nurses, significantly fewer doctors reported the use of CPGs in their clinical practice (127/295, 43.1% vs. 177/236, 75.0%; % difference −31.9%, 95% CI −39.6 to −23.8%), whereas a higher proportion of doctors reported providing patient information sheets (222/296, 75.0% vs. 163/238, 68.5%; % difference 6.5%, 95% CI −1.2% to 14.2%) (Table 2).

Clinicians working in specialist paediatric EDs were more likely to utilise both CPGs (125/201, 62.2% vs. 161/303, 53.1%; % difference 9.1%, 95% CI 0.2%–17.7%) and patient information sheets (162/202, 80.2% vs. 201/304, 66.1%; % difference 14.1%, 95% CI 6.2%–21.5%) than those in general ED settings (Table 3).

#### 3.2.4 Antipyretic Use for Febrile Convulsions

As illustrated in Table 5a, slightly over one‐third of respondents indicated that they would give antipyretics for febrile convulsion prevention during the current illness. Compared to nurses, a significantly smaller proportion of doctors (66/296, 22.3% vs. 126/237, 53.2%; % difference −30.9%, 95% CI −38.6% to −22.7%) would encourage antipyretic use for this indication.

Additionally, clinicians from specialist paediatric EDs reported lower rates of antipyretic use for convulsion prevention compared to their counterparts in general EDs (58/201, 28.9% vs. 121/304, 39.8%; % difference −10.9%, 95% CI −19.1% to −2.5%) (Table 3).

## 4 Discussion

Current international consensus guidelines recommend antipyretic therapy in febrile children primarily for discomfort relief, instead of solely for temperature reduction [3, 8]. Our study demonstrates that only 9.3% of Australian ED clinicians self‐reported adherence to these recommendations. Given how common febrile children are in the ED setting, this is likely leading to overuse of antipyretic medications in hospitals, and potentially contributes to worsening fever phobia in parents/caregivers.

The low adherence to international consensus recommendations observed in our study aligns with previously published findings from the New Zealand study, with only 10.6% of respondents demonstrating adherence to best practice recommendations [12]. New Zealand and Australian ED clinicians are not unique; in Turkey, only 15% of surveyed physicians considered the relief of discomfort when giving antipyretics for fever management [6], a consideration similarly noted by just 38% of Italian paediatricians [11] and 64% of Swiss paediatricians [19]. In our cohort, over 80% of participants reported administering antipyretics primarily to reduce temperature, a practice which is also seen in 74% of New Zealand ED clinicians [12]; similar sentiments have been identified in around two‐thirds of paediatric ED nurses in Portugal [20], 39% of Australian paediatric nurses [21] and nearly 75% of Turkish doctors [6].

Nearly half of our participants self‐reported recommending a combined antipyretic regimen, in contrast to just 28% of ED clinicians in New Zealand [12]. Combined antipyretic therapy may reduce fever slightly more than monotherapy (around 0.3°C) [22, 23]. However, the clinical significance of this is questionable [22, 23] and safety concerns [24] have led to this approach not being recommended [3, 8].

While guidelines in New Zealand are consistent with international best practice recommendations, clinical practice guidelines in Australia do not routinely comment on ideal antipyretic use for febrile infants (rather, focusing on risk stratification for sepsis) and some parental advice sheets do not appear to agree with international consensus (instead suggesting combined antipyretic use).

Over 50% of our participants reported using clinical practice guidelines for antipyretic administration in febrile presentations, and approximately three‐quarters indicated routinely providing caregivers with informational materials at discharge—findings similar to those reported in the New Zealand study [12]. While these figures suggest a broadly positive engagement with evidence‐based care and caregiver education, variation in care is evident. This variation, along with mixed messages from clinician statements, clinician behaviour and written advice sheets may exacerbate caregiver anxiety, undermine trust in medical advice and contribute to improper antipyretic use [13].

This study also identified an increased likelihood of antipyretic use in the setting of abnormal vital signs among otherwise settled infants, similar to that observed in the New Zealand study [12]. Although best practice guidelines caution against antipyretic use solely for physiological abnormalities such as elevated heart or respiratory rates [8], vital sign deviations remain important clinical markers of potential serious infection and have been incorporated into validated risk‐assessment tools [8, 25]. The tendency to attempt to normalise vital signs through antipyretic intervention likely reflects a desire to improve clinical stability and mitigate concerns around potential serious illness [12]. However, this approach may be misguided, as reliance on vital sign normalisation alone is not recommended for distinguishing serious bacterial infections [8]. Notably, while persistent tachypnoea following antipyretic administration may support a diagnosis of pneumonia, persistent tachycardia alone lacks a clear association with increased serious bacterial infection risks [26]. In light of this, further research is warranted to determine if clinical assessment following antipyretic use improves the diagnostic accuracy of serious illness in febrile children [8].

Over one‐third of our respondents recommended antipyretic use to prevent febrile convulsions during the current illness, mirroring previous studies that reported such practices in 76% of primary care physicians [6] and 57% of ED clinicians [12]. Notably, nurses exhibited more than twice the likelihood of endorsing this approach compared to doctors. Erroneous beliefs among healthcare providers regarding the associated risks of untreated fever, particularly febrile convulsions as highlighted by multiple studies [6, 20, 21], may drive the overuse of antipyretics. While a randomised controlled trial from Japan suggested that regular paracetamol use compared to no paracetamol use reduced the risk of recurrent febrile convulsions within the same febrile episode [27], prevailing evidence from prior systematic reviews still indicates limited clinical benefit [28]. Further high‐quality research is warranted before any changes to current clinical practice guidelines, which continue to discourage prophylactic antipyretics for this purpose [3, 8].

Reasons for clinicians aggressively treating fever (a normal response to infection), particularly in the presence of accompanying tachycardia and tachypnoea, require further exploration. Clear definition and measurement of febrile discomfort would allow a more targeted approach to therapy. Explicit recommendations on antipyretic therapy in commonly used clinical guidelines, accompanied by national and local knowledge translation efforts addressing relevant barriers and facilitators, may also reduce the practice variation evident in our results.

Strengths of this study include its large, diverse sample size drawn from multiple varied ED settings reflecting real‐world clinical practices. Key limitations of this study include potential responder bias due to moderate overall response rates (65.9%). We did not account for clustering by site in our analysis. The use of clinical vignettes may introduce social desirability bias, where participants report based on their clinical guideline knowledge [12]. The cross‐sectional design restricts causal interpretations, and the lack of post hoc analyses constrains deeper examination of how clinician characteristics influence practice.

## 5 Conclusion

Less than 10% of Australian ED clinicians self‐report adherence to international evidence‐based clinical practice guidelines for antipyretic use in paediatric fever management. Given the high volume of febrile children presenting to EDs each year, updates to local clinical practice guidelines may reduce variation in care and improve the quality and consistency of paediatric fever management.

## Conflicts of Interest

Simon Craig and Shane George are section editors of Emergency Medicine Australasia.

## Supporting information


**Table S1:** Antipyretic use among doctors and nurses based on clinical vignette patients with a normal heart rate and respiratory rate, and variable levels of discomfort and fluid intake.
**Table S2:** Antipyretic use among doctors and nurses based on clinical vignette patients with an elevated heart rate and respiratory rate, and variable levels of discomfort and fluid intake.

## Data Availability

The data that support the findings of this study are available from the corresponding author upon reasonable request.
