# Supplementary material for: Paediatric Fever Management Practices and Antipyretic Use Among Doctors and Nurses in Australian Emergency Departments
Source: Emerg Med Australas. 2025 Nov 3;37(6):e70165. doi: 10.1111/1742-6723.70165 (PMC12580986; doi:10.1111/1742-6723.70165)
Supplement: Supplementary file 1 — Table S1: Antipyretic use among doctors and nurses based on clinical vignette patients with a normal heart rate and respiratory rate, and variable levels of discomfort and fluid intake. Table S2: Antipyretic use among doctors and nurses based on clinical vignette patients with an elevated heart rate and respiratory rate, and variable levels of discomfort and fluid intake. [file EMM-37-0-s001.docx]

**Supporting Information**

**Appendix 1 – Survey questionnaire**

**Paediatric fever and paracetamol/ibuprofen use – perspectives and practice patterns among doctors and nurses in Australian emergency departments**

The aim of this survey is to describe the perspectives and practice patterns among Australian emergency department (ED) nurses and doctors in relation to fever and use of paracetamol and ibuprofen in children <2 years of age.

**Inclusion and exclusion criteria**

**Inclusion criteria** (please tick all that apply – need both criteria to participate):

- Full or part-time ED doctor or nurse.
- Work regularly (at least 1 shift per week, on average) in an ED that sees children <2 years of age.

**Exclusion criteria** (please tick all that apply – need to have none of these to participate):

- Not currently engaged in clinical practice.
- Junior Medical Staff rotating through ED placement.
- Medical locum or nursing agency staff.
- Medical or nursing student.

**Participant information summary**

All information that you provide will be **anonymous**. No information which could personally identify you will be used in any reports from this study. The aim of this survey is to describe the practice patterns for groups of nurses and doctors. We will **not** examine responses of individuals or any small subgroups that might implicitly identify individuals.

Please take a few moments to read the Participant Information Sheet.

**Consent**

I have read the Participant Information Sheet and I agree to take part in this study.

- Yes
- No

**Start of survey**

Thank you for agreeing to complete this survey.

Please read each question carefully. We are interested in YOUR views and what you would do in your USUAL practice. There are no correct or incorrect responses.

The survey is in four parts and will take approximately 10-15 minutes to complete.

**Part A. Clinical scenarios**

**Scenario 1-3**

A 6-month-old infant presents to your ED with a chief complaint of fever for the past 24 hours. There are no other associated symptoms. There is no significant past medical history. The infant is not on any regular medications and has no allergies.

T 39°C (tympanic), HR 140/min, RR 30/min, SaO_2_ 98%, weight 8kg.

| **Scenario** | **Oral intake** | **Examination findings** | **With regards to paracetamol and/or ibuprofen, what would you do for this patient?** |
| --- | --- | --- | --- |
| 1 | The infant has been **drinking normally**. | The **infant is** **settled** with Mum.  The infant is fixing and following.  No focus for fever found on examination. | - Give paracetamol - Give ibuprofen - Give either paracetamol OR ibuprofen (it does not matter which) - Give both paracetamol AND ibuprofen at the same time - I will not give any paracetamol or ibuprofen at this time |
| 2 | The infant has been **drinking normally**. | The **infant is crying intermittently**, but is able to settle with Mum.  The infant is fixing and following.  No focus for fever found on examination. | - Give paracetamol - Give ibuprofen - Give either paracetamol OR ibuprofen (it does not matter which) - Give both paracetamol AND ibuprofen at the same time - I will not give any paracetamol or ibuprofen at this time |
| 3 | The infant has been **drinking about two-thirds** of the usual amount. | The **infant is** **settled** with Mum.  The infant is fixing and following.  No focus for fever found on examination. | - Give paracetamol - Give ibuprofen - Give either paracetamol OR ibuprofen (it does not matter which) - Give both paracetamol AND ibuprofen at the same time - I will not give any paracetamol or ibuprofen at this time |

**Scenario 4-6**

A 6-month-old infant presents to your ED with a chief complaint of fever for the past 24 hours. There are no other associated symptoms. There is no significant past medical history. The infant is not on any regular medications and has no allergies.

T 39°C (tympanic), **HR 180/min**, **RR 50/min**, SaO_2_ 98%, weight 8kg.

| **Scenario** | **Oral intake** | **Examination findings** | **With regards to paracetamol and/or ibuprofen, what would you do for this patient?** |
| --- | --- | --- | --- |
| 4 | The infant has been **drinking normally**. | The **infant is settled** with Mum.  The infant is fixing and following.  No focus for fever found on examination. | - Give paracetamol - Give ibuprofen - Give either paracetamol OR ibuprofen (it does not matter which) - Give both paracetamol AND ibuprofen at the same time - I will not give any paracetamol or ibuprofen at this time |
| 5 | The infant has been **drinking normally**. | The **infant is crying intermittently**, but is able to settle with Mum.  The infant is fixing and following.  No focus for fever found on examination. | - Give paracetamol - Give ibuprofen - Give either paracetamol OR ibuprofen (it does not matter which) - Give both paracetamol AND ibuprofen at the same time - I will not give any paracetamol or ibuprofen at this time |
| 6 | The infant has been **drinking about two-thirds** of the usual amount. | The **infant is settled** with Mum.  The infant is fixing and following.  No focus for fever found on examination. | - Give paracetamol - Give ibuprofen - Give either paracetamol OR ibuprofen (it does not matter which) - Give both paracetamol AND ibuprofen at the same time - I will not give any paracetamol or ibuprofen at this time |

**Scenario 7**

A 6-month-old infant presents to your ED with a chief complaint of fever for the past 24 hours. There are no other associated symptoms. There is no significant past medical history. The infant is not on any regular medications and has no allergies.

T 39°C (tympanic), HR 140/min, RR 30/min, SaO_2_ 98%, weight 8kg.

The infant has been drinking normally. The infant is settled with Mum. The infant is fixing and following. No focus for fever is found on examination.

A urine test is normal and you decide to discharge with a diagnosis of fever secondary to viral illness.

The parent/caregiver asks for advice regarding paracetamol and/or ibuprofen if their infant becomes **hot** and **unsettled** again?

With regards to paracetamol and/or ibuprofen, what advice would you give the parent/caregiver?

- Give either paracetamol OR ibuprofen if the infant becomes hot and unsettled, repeating doses of the same medication if the infant remains hot and unsettled.
- Give either paracetamol OR ibuprofen if the infant becomes hot and unsettled, alternating with the other medication if the infant remains hot and unsettled.
- Give both paracetamol and ibuprofen at the same time if the infant becomes hot and unsettled.
- There is no need to give any paracetamol or ibuprofen in this situation (viral illness) because the fever will settle.

**Scenario 8**

A 12-month-old infant presents to your ED with a chief complaint of seizure. The infant has had a runny nose for the past two days, but has been eating and drinking normally. The history of the seizure is consistent with a simple febrile seizure which lasted less than one minute. After a ten-minute post-ictal period, the infant is now back to their usual self. There is no significant past medical history. The infant is not on any regular medications and has no allergies. On examination, vital signs are:

T 39°C (tympanic), HR 130/min, RR 25/min, SaO_2_ 98%, weight 10kg.

The infant is settled with Mum and is playing with toys. Other than clear coryza, there is no other focus for the fever on examination.

With regards to **prevention of febrile convulsions** during **this illness**, what discharge advice would you give the parent/caregiver about paracetamol and/or ibuprofen?

| **Initial options** | **Branching logic** |
| --- | --- |
| - Give **medications** **regularly** to prevent febrile convulsions | What would you advise to give first?   - Paracetamol - Ibuprofen - Either paracetamol OR ibuprofen (it does not matter which) - Both paracetamol AND ibuprofen at the same time |
| - Give **medications** **as needed** if there is a fever to prevent febrile convulsions | What would you advise to give first?   - Paracetamol - Ibuprofen - Either paracetamol OR ibuprofen (it does not matter which) - Both paracetamol AND ibuprofen at the same time |
| - I would advise that paracetamol and/or ibuprofen are not required to prevent febrile convulsions | -- |

With regards to **management of fever** during **this illness**, what discharge advice would you give the parent/caregiver about paracetamol and/or ibuprofen?

| **Initial options** | **Branching logic** |
| --- | --- |
| - Give **medications regularly** to prevent fever | What would you advise to give first?   - Paracetamol - Ibuprofen - Either paracetamol OR ibuprofen (it does not matter which) - Both paracetamol AND ibuprofen at the same time |
| - Give **medications as needed** if there is a fever | What would you advise to give first?   - Paracetamol - Ibuprofen - Either paracetamol OR ibuprofen (it does not matter which) - Both paracetamol AND ibuprofen at the same time |
| - Give **medications as needed** if there is a fever and the infant is distressed | What would you advise to give first?   - Paracetamol - Ibuprofen - Either paracetamol OR ibuprofen (it does not matter which) - Both paracetamol AND ibuprofen at the same time |
| - Give **medications as needed** if the infant is distressed, regardless of fever | What would you advise to give first?   - Paracetamol - Ibuprofen - Either paracetamol OR ibuprofen (it does not matter which) - Both paracetamol AND ibuprofen at the same time |
| - I would advise that the use of paracetamol and/or ibuprofen is not needed at this time | -- |

**9. Do you follow clinical practice guidelines when using paracetamol and/or ibuprofen for febrile children in the ED?**

| **Initial options** | **Branching logic** |
| --- | --- |
| - No | -- |
| - Yes | Which guidelines do you follow? (Tick ALL that apply)   - Local hospital - State guidelines (please specify state) - Royal Children’s Hospital - National Institute for Health and Care Excellence (NICE) - American Academy of Pediatrics - Other: ____________________________________(please specify) |

**10. Do you give parents/caregivers an information sheet about fever when discharging febrile children from the ED?**

| **Initial options** | **Branching logic** |
| --- | --- |
| - No | -- |
| - Yes | Which information sheet do you use? (Tick ALL that apply)   - Local hospital - Royal Children’s Hospital - Kids Health Information - State Guidelines (please specify state) - Better Health Channel - Health Direct - Patient.info (UK) - UpToDate - Other: **____________________________________** (please specify) |

**Part B. Factors influencing fever management and paracetamol/ibuprofen use**

The following questions relate to the factors that influence your use of paracetamol and/or ibuprofen in febrile children <2 years of age. Please rate each statement by choosing the option that best reflects YOUR views or usual practice.

|  | **Strongly disagree** | **Disagree** | **Neutral** | **Agree** | **Strongly agree** |
| --- | --- | --- | --- | --- | --- |
| I know the content and objectives of clinical practice guidelines regarding paracetamol and/or ibuprofen use in febrile children. | 1 | 2 | 3 | 4 | 5 |
| I have been trained to ensure paracetamol and/or ibuprofen are given to febrile children only if they appear distressed. | 1 | 2 | 3 | 4 | 5 |
| I have been trained to identify distress in febrile children less than 2 years old. | 1 | 2 | 3 | 4 | 5 |
| Giving paracetamol and/or ibuprofen to febrile children only if they appear distressed is part of my work as an ED doctor/nurse. | 1 | 2 | 3 | 4 | 5 |
| I feel confident explaining to parents/caregivers that paracetamol and/or ibuprofen are used for fever only if their child appears distressed. | 1 | 2 | 3 | 4 | 5 |
| I have control over ensuring paracetamol and/or ibuprofen are given to febrile children only if they appear distressed. | 1 | 2 | 3 | 4 | 5 |
| For me, giving paracetamol and/or ibuprofen to febrile children only if they appear distressed may increase the risk of convulsions in non-distressed febrile children. | 1 | 2 | 3 | 4 | 5 |
| When managing febrile children in ED, my aim is to reduce the fever before discharge. | 1 | 2 | 3 | 4 | 5 |
| In the ED I work in, giving paracetamol and/or ibuprofen to febrile children only if they appear distressed is routine/the standard of care. | 1 | 2 | 3 | 4 | 5 |
| I am confident that I can ensure paracetamol and/or ibuprofen are given to febrile children only if they appear distressed, even when the ED is busy. | 1 | 2 | 3 | 4 | 5 |
| In the ED, I have time to educate parents/caregivers about fever and use of paracetamol and/or ibuprofen only if their child appears distressed. | 1 | 2 | 3 | 4 | 5 |
| I feel challenged/pressured by parents/caregivers of children to intervene and give paracetamol and/or ibuprofen to reduce fever regardless of distress. | 1 | 2 | 3 | 4 | 5 |
| I feel challenged/pressured by colleagues to intervene and give paracetamol and/or ibuprofen to reduce fever regardless of the child’s distress. | 1 | 2 | 3 | 4 | 5 |
| Most colleagues whose opinion I value would approve of me giving paracetamol and/or ibuprofen to febrile children only if they appear distressed. | 1 | 2 | 3 | 4 | 5 |

**Part C. Response to paracetamol and/or ibuprofen in febrile children**

**1.** When using paracetamol and/or ibuprofen for febrile children in the ED, which of the following clinical features would you consider important in determining whether or not they had a *satisfactory response to antipyretics?* **(select ALL that apply**)

| **Clinical outcome** |  |  |
| --- | --- | --- |
| Reduction in valid pain/distress score to “no pain/distress”  Reduction in valid pain/distress score, but not necessarily to “no pain/distress”  Reduction in temperature to normal range |  |  |
| Reduction in temperature, but not necessarily to normal range |  |  |
| Reduction in heart rate to normal range  Reduction in heart rate, but not necessarily to normal range |  |  |
| Reduction in respiratory rate to normal range |  |  |
| Reduction in respiratory rate, but not necessarily to normal range |  |  |
| Improvement in lethargy/irritability and back to usual behaviour  Improvement in lethargy/irritability, but not necessarily to usual behaviour |  |  |
| Improvement in fluid intake to normal intake |  |  |
| Improvement in fluid intake, but not necessarily to normal intake |  |  |
| Other: ________________ |  |  |

**2.** When using paracetamol and/or ibuprofen for febrile children in the ED, which clinical feature do you consider most indicative of a satisfactory response to antipyretics?

**(Select the MOST important outcome)**

| **Clinical outcome** |  |  |
| --- | --- | --- |
| Reduction in valid pain/distress score to “no pain/distress”  Reduction in valid pain/distress score, but not necessarily to “no pain/distress”  Reduction in temperature to normal range |  |  |
| Reduction in temperature, but not necessarily to normal range |  |  |
| Reduction in heart rate to normal range  Reduction in heart rate, but not necessarily to normal range |  |  |
| Reduction in respiratory rate to normal range |  |  |
| Reduction in respiratory rate, but not necessarily to normal range |  |  |
| Improvement in lethargy/irritability and back to usual behaviour  Improvement in lethargy/irritability, but not necessarily to usual behaviour |  |  |
| Improvement in fluid intake to normal intake |  |  |
| Improvement in fluid intake, but not necessarily to normal intake |  |  |
| Other: ________________ |  |  |

**Part D. Demographic data**

1. **Which hospital do you work at the majority (>50%) of the time?**

- __________________________

1. **What is your profession?**

| **Initial options** | **Branching logic** |
| --- | --- |
| - Doctor | What is your clinical role?   - Consultant - Fellow - Registrar - Other: ___________________________ |
| - Nurse | What is your clinical role?   - Advanced practice role (Nurse Specialist, Nurse Practitioner) - Senior nurse (Clinical Educator, Charge) - Registered nurse - Enrolled nurse - Other: ___________________________ |

1. **How long have you been working as a nurse or a doctor?**

- 0-4 years
- 5-9 years
- 10-14 years
- ≥15 years

1. **Do you have paediatric-specific qualifications (e.g. Nursing Masters, Diploma of Paediatrics, FRACP, sub-speciality ACEM training in PEM)?**

- Yes
- No

***﻿Thank you for taking the time to complete this survey***

**Appendix 2 - Primary outcome measure of adherence to fever management best practice guidelines as demonstrated by response to four vignettes with different vital signs and levels of discomfort in a 6-month-old infant.** Sourced from (12)

| **Discomfort level** | **Vital signs** | |
| --- | --- | --- |
|  | T 39°C (tympanic)  HR 140/min, RR 30/min | T 39°C (tympanic)  HR 180/min, RR 50/min |
| Settled, drinking usual amount  (Discomfort not present) | Best practice: **I will not give paracetamol or ibuprofen at this time** | Best practice: **I will not give paracetamol or ibuprofen at this time** |
| Crying intermittently, drinking usual amount  (Discomfort present) | Best practice: **Give paracetamol OR**  **Give ibuprofen OR**  **Give either paracetamol or ibuprofen (it does not matter which)** | Best practice: **Give paracetamol OR**  **Give ibuprofen OR**  **Give either paracetamol or ibuprofen (it does not matter which)** |

2 x 2 table shows the combination of vital signs and discomfort levels described in the four vignettes (Appendix 1 – Scenario 1, 2, 4 and 5). The primary outcome of adherence was defined as single antipyretic use for the relief of patient discomfort, rather than for the sole purpose of temperature reduction. The primary outcome measure was met if participants responded to all four vignettes as indicated in bold when presented with a tick-box question: *"with regards to paracetamol and/or ibuprofen, what would you do for this patient?"*

HR, heart rate; RR, respiratory rate; T, temperature.

**Supplementary TABLE 1.** Antipyretic use among doctors and nurses based on clinical vignette patients with a normal heart rate and respiratory rate, and variable levels of discomfort and fluid intake.

| **Clinical vignette** | **All participants** | | **Doctors** | | **Nurses** | | **Absolute difference in proportion (%)**  **(95% CI)** | ***P*** |
| --- | --- | --- | --- | --- | --- | --- | --- | --- |
|  | ***N*** | ***n* (%, % 95 CI)** | ***N*** | ***n* (%, % 95 CI)** | ***N*** | ***n* (%, 95% CI)** |  |  |
| **Settled, drinking usual amount** | 539 |  | 300 |  | 239 |  |  |  |
| Give a single antipyretic (paracetamol or ibuprofen) |  | 163 (30.2, 26.5-34.2) |  | 98 (32.7, 27.6-38.2) |  | 65 (27.2, 21.9-33.2) | 5.5 (-2.3-13.1) | 0.17 |
| Give both paracetamol and ibuprofen at the same time |  | 55 (10.2, 7.9-13.1) |  | 39 (13.0, 9.6-17.3) |  | 16 (6.7, 4.1-10.7) | 6.3 (1.2-11.2) | 0.02 |
| Paracetamol and/or ibuprofen not needed at this time |  | 321 (59.6, 55.4-63.6) |  | 163 (54.3, 48.7-59.9) |  | 158 (66.1, 59.9-71.9) | -11.8 (-19.9 to -3.5) | 0.01 |
| **Settled, drinking 2/3 usual amount^†^** | 538 |  | 300 |  | 238 |  |  |  |
| Give a single antipyretic (paracetamol or ibuprofen) |  | 287 (53.3, 49.1-57.5) |  | 178 (59.3, 53.7-64.7) |  | 109 (45.8, 39.6-52.1) | 13.5 (5.0-21.8) | <0.01 |
| Give both paracetamol and ibuprofen at the same time |  | 101 (18.8, 15.7-22.3) |  | 66 (22.0, 17.7-27.0) |  | 35 (14.7, 10.7-19.8) | 7.3 (0.7-13.7) | 0.03 |
| Paracetamol and/or ibuprofen not needed at this time |  | 150 (27.9, 24.3-31.8) |  | 56 (18.7, 14.6-23.5) |  | 94 (39.5, 33.5-45.8) | -20.8 (-28.3 to -13.1) | <0.01 |
| **Crying intermittently, drinking usual amount** | 539 |  | 300 |  | 239 |  |  |  |
| Give a single antipyretic (paracetamol or ibuprofen) |  | 339 (62.9, 58.7-66.9) |  | 200 (66.7, 61.1-71.8) |  | 139 (58.2, 51.8-64.2) | 8.5 (0.3-16.7) | 0.04 |
| Give both paracetamol and ibuprofen at the same time |  | 102 (18.9, 15.8-22.5) |  | 72 (24.0, 19.5-29.2) |  | 30 (12.6, 8.9-17.4) | 11.4 (4.9-17.7) | <0.01 |
| Paracetamol and/or ibuprofen not needed at this time |  | 98 (18.2, 15.1-21.7) |  | 28 (9.3, 6.5-13.2) |  | 70 (29.3, 23.9-35.4) | -20.0 (-26.5 to -13.2) | <0.01 |
| † Data missing for the following question: settled, drinking 2/3 usual amount (n=1).  CI, confidence interval. | | | | | | | | |

**Supplementary TABLE 2.** Antipyretic use among doctors and nurses based on clinical vignette patients with an elevated heart rate and respiratory rate, and variable levels of discomfort and fluid intake.

| **Clinical vignette** | **All participants** | | **Doctors** | | **Nurses** | | **Absolute difference in proportion (%)**  **(95% CI)** | ***P*** |
| --- | --- | --- | --- | --- | --- | --- | --- | --- |
|  | ***N*** | ***n* (%, % 95 CI)** | ***N*** | ***n* (%, % 95 CI)** | ***N*** | ***n* (%, 95% CI)** |  |  |
| **Settled, drinking usual amount** | 539 |  | 300 |  | 239 |  |  |  |
| Give a single antipyretic (paracetamol or ibuprofen) |  | 277 (51.4, 47.2-55.6) |  | 165 (55.0, 49.3-60.5) |  | 112 (46.9, 40.6-53.2) | 8.1 (-0.4-16.5) | 0.06 |
| Give both paracetamol and ibuprofen at the same time |  | 171 (31.7, 27.9-35.8) |  | 104 (34.7, 29.5-40.2) |  | 67 (28.0, 22.7-34.1) | 6.6 (-1.3-14.4) | 0.10 |
| Paracetamol and/or ibuprofen not needed at this time |  | 91 (16.9, 13.9-20.3) |  | 31 (10.3, 7.3-14.3) |  | 60 (25.1, 20.0-31.0) | -14.8 (-21.2 to -8.2) | <0.01 |
| **Settled, drinking 2/3 usual amount^†^** | 535 |  | 298 |  | 237 |  |  |  |
| Give a single antipyretic (paracetamol or ibuprofen) |  | 290 (54.2, 50.0-58.4) |  | 176 (59.1, 53.4-64.5) |  | 114 (48.1, 41.8-54.4) | 11.0 (2.5-19.3) | 0.01 |
| Give both paracetamol and ibuprofen at the same time |  | 164 (30.7, 26.9-34.7) |  | 97 (32.6, 27.5-38.1) |  | 67 (28.3, 22.9-34.3) | 4.3 (-3.6-12.0) | 0.29 |
| Paracetamol and/or ibuprofen not needed at this time |  | 81 (15.1, 12.3-18.4) |  | 25 (8.4, 5.7-12.1) |  | 56 (23.6, 18.7-29.4) | -15.2 (-21.5 to -8.9) | <0.01 |
| **Crying intermittently, drinking usual amount** | 539 |  | 300 |  | 239 |  |  |  |
| Give a single antipyretic (paracetamol or ibuprofen) |  | 295 (54.7, 50.5-58.9) |  | 173 (57.7, 52.0-63.1) |  | 122 (51.0, 44.7-57.3) | 6.6 (-1.8-15.0) | 0.13 |
| Give both paracetamol and ibuprofen at the same time |  | 187 (34.7, 30.8-38.8) |  | 115 (38.3, 33.0-44.0) |  | 72 (30.1, 24.7-36.2) | 8.2 (0.1-16.1) | 0.05 |
| Paracetamol and/or ibuprofen not needed at this time |  | 57 (10.6, 8.2-13.5) |  | 12 (4.0, 2.2-6.7) |  | 45 (18.8, 14.4-24.3) | -14.8 (-20.2 to -9.3) | <0.01 |
| † Data missing for the following question: settled, drinking 2/3 usual amount (n=4).  CI, confidence interval. | | | | | | | | |
